# The Lemon Flavonoid Eriomin^®^ Suppresses Pituitary–Adrenal Axis Activity in Aged Rats

**DOI:** 10.3390/ijms26125818

**Published:** 2025-06-17

**Authors:** Svetlana Trifunović, Ivona Gizdović, Nataša Ristić, Branko Filipović, Vladimir Ajdžanović, Marko Miler, Thais Cesar, Branka Šošić-Jurjević

**Affiliations:** 1 Institute for Biological Research “Siniša Stanković”—National Institute of Republic of Serbia, University of Belgrade, Bulevar despota Stefana 142, 11108 Belgrade, Serbia; ivona.gizdovic@ibiss.bg.ac.rs (I.G.); negicn@ibiss.bg.ac.rs (N.R.); brankof@ibiss.bg.ac.rs (B.F.); avlada@ibiss.bg.ac.rs (V.A.); marko.miler@ibiss.bg.ac.rs (M.M.); brankasj@ibiss.bg.ac.rs (B.Š.-J.); 2 Graduate Program in Food, Nutrition and Food Engineering, Sao Paulo State University (UNESP), Araraquara 14800-060, Brazil; thais.cesar@unesp.br

**Keywords:** lemon flavonoid extract Eriomin^®^, pituitary gland, adrenal gland, aged rat, healthy ageing

## Abstract

The lemon flavonoid extract Eriomin^®^ (LE), which is rich in eriocitrin, has demonstrated antioxidant and anti-inflammatory properties in both animal and human studies. Given the established interplay among aging, oxidative stress, and inflammation, this study investigated the influences of LE on the pituitary–adrenal (PA) axis in aged rats and its potential to mitigate age-related physiological changes in this system. The effects of LE (40 mg/kg/day suspended in sunflower oil) on the morphofunctional properties of the PA axis were studied in 24-month-old male Wistar rats following four weeks of oral treatment. Control groups included vehicle-treated (sunflower oil; CON) and untreated intact controls (ICON). Stereological and imaging analyses revealed no significant changes in pituitary ACTH cells; however, *Pomc* gene expression was significantly downregulated in the LE group compared to both controls (*p* ≤ 0.05). LE treatment resulted in a significant reduction in adrenal gland weight (*p* ≤ 0.05), adrenal gland volume (*p* ≤ 0.01), zona fasciculata (ZF) volume (*p* ≤ 0.01) and ZF cell volume (*p* ≤ 0.05). These changes were accompanied by a significant decrease in serum corticosterone levels (*p* ≤ 0.05). In conclusion, LE downregulated PA axis activity in aged rats. Considering the association between age-related increases in PA activity and adverse health outcomes, citrus flavonoid extracts such as LE may hold promise as anti-aging supplements aimed at mitigating age-related stress dysregulation.

## 1. Introduction

Aging is a continuous and irreversible process characterized by a gradual decline in physiological functions and an increased risk of various diseases, including metabolic, stress-related, cognitive, cardiovascular, and neurodegenerative disorders. According to the World Health Organization, by 2030, the proportion of the global population aged 65 and older will be greater than the proportions of younger age groups. Consequently, research aimed at preventing or delaying the onset of age-related disabilities to promote healthy aging is becoming increasingly vital [1,2].

During aging, hormone secretory patterns and sensitivity to negative feedback by end hormones change, affecting the regulation and maintenance of physiological homeostasis [3]. At the level of the pituitary–adrenal (PA) axis (as the functional module of the hypothalamic–pituitary–adrenal axis), aging is associated with increased cortisol/corticosterone levels, disruption of negative feedback by glucocorticoids (GC), and attenuation of the diurnal rhythm of GC secretion [4], while ACTH levels remain relatively stable [3]. Age-related dysfunction of the PA axis under both basal and stress conditions disrupts key physiological functions, such as metabolism and cardiovascular regulation, thereby contributing to pathophysiological conditions that favor the development of age-related diseases [5,6]. The interplay among increased PA axis activity, oxidative stress, and inflammation during aging forms a self-reinforcing cycle that accelerates age-related physiological decline. Thus, the age-associated elevation in basal cortisol levels is partly due to hippocampal damage caused by oxidative stress, which impairs cortisol regulation [7]. Conversely, chronic cortisol elevation contributes to oxidative damage in different tissues and may play pivotal roles in age-related pathology [8,9,10].

**Figure 1:**
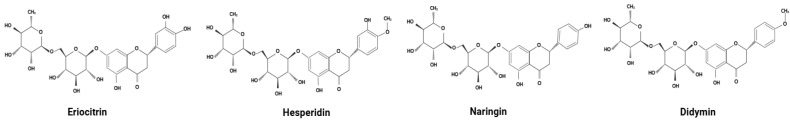
Chemical structures of the main citrus flavonoids in Eriomin^®^.

There is growing evidence that certain fruits have significant health benefits, and research into natural compounds that promote healthy aging by maintaining metabolic and endocrine balance—which is crucial for overall physiological homeostasis—is highly valuable. The lemon flavonoid extract Eriomin^®^ (LE), which is mainly composed of eriocitrin, hesperidin, naringin, and didymin (chemical structures are presented in Figure 1), has been recognized for its anti-inflammatory, antioxidant (the ortho-hydroxyl groups on the B ring of eriocitrin indicate antioxidant potential), antitumor, and antidiabetic properties [11,12].

An earlier study demonstrated that LE supplementation can reduce the excessive oxidative stress associated with diabetes mellitus and other chronic diseases [13,14,15]. Supporting this, our previous studies showed that naringenin and hesperetin exert antioxidant effects in the liver and in the pituitary and thyroid glands of aged animal models [16,17,18]. Our latest research revealed that LE acts as a potent antioxidant by effectively reducing oxidative stress in the small intestine and the liver through the modulation of the hepatic redox regulators Nrf2 and Trx1, as well as through alterations in protein persulfidation levels [19,20].

Considering the beneficial effects of citrus flavonoids in reducing oxidative stress and the established links among aging, oxidative stress, and elevated cortisol levels, we aimed to investigate the influence of LE on age-related imbalances in the PA axis. This investigation included a comprehensive analysis of histomorphological changes in pituitary ACTH-immunoreactive cells and *Pomc* gene expression, alongside assessments of the adrenal cortex, with particular focus on the zona fasciculata (ZF), the primary site of corticosterone production in aging rats.

## 2. Results

### 2.1. Body Mass, Pituitary, and Adrenal Gland Weight

The results for body mass and the absolute and relative weights of the pituitary and adrenal glands are shown in Table 1. In the LE-treated group, the absolute and relative weights of the adrenal gland decreased by 18% (*p* ≤ 0.001) and 20% (*p* ≤ 0.05), respectively, compared to those in the CON group.

### 2.2. Histological, Stereological, and Molecular Insights into Pituitary ACTH Cells

**Figure 2:**
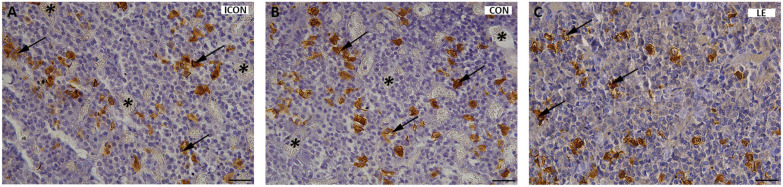
ACTH immunopositivity in the pituitary gland of aged rats in the intact control group (ICON, **A**), the sunflower-oil control group (CON, **B**), and the group treated with the lemon flavonoid extract Eriomin^®^ (LE, **C**). ACTH cells (arrows), blood vessels (asterisks); scale bar = 20 μm.

In all experimental groups, ACTH-immunopositive cells in the anterior pituitary were observed either individually or in groups between capillaries in the pars distalis. These cells exhibited multiple morphologies, ranging from oval to polygonal in shape, with some displaying strong elongated projections extending toward neighboring cells. Histological analysis revealed no changes in the shape, frequency, or distribution of ACTH cells among the groups (Figure 2A–C). Moreover, no visible histopathological changes were detected upon LE treatment.

**Figure 3:**
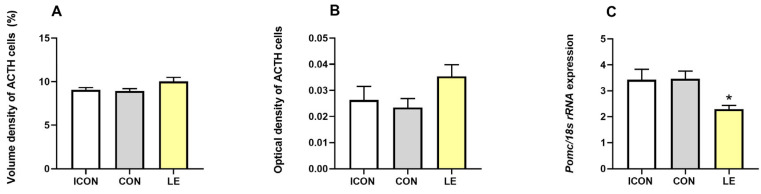
Volume (**A**) and optical density (**B**) of ACTH cells and *Pomc* mRNA expression (**C**) in aged rats in the intact control group (ICON), the sunflower-oil control group (CON) and the lemon flavonoid extract Eriomin^®^ group (LE). Data are presented as mean ± SEM (*n* = 6/group); one-way ANOVA test followed by Dunnett’s post hoc test (with CON serving as the control group); * *p* ≤ 0.05.

Stereological and image analyses showed no differences in the volume or optical density of ACTH cells between groups (Figure 3A,B). However, mRNA expression of proopiomelanocortin (*Pomc*), the precursor of ACTH, was decreased by 34% in the LE group compared to the CON group, and this difference was significant (*p* ≤ 0.05) (Figure 3C).

### 2.3. Histological, Stereological, and Biochemical Insights into the Adrenal Gland

**Figure 4:**
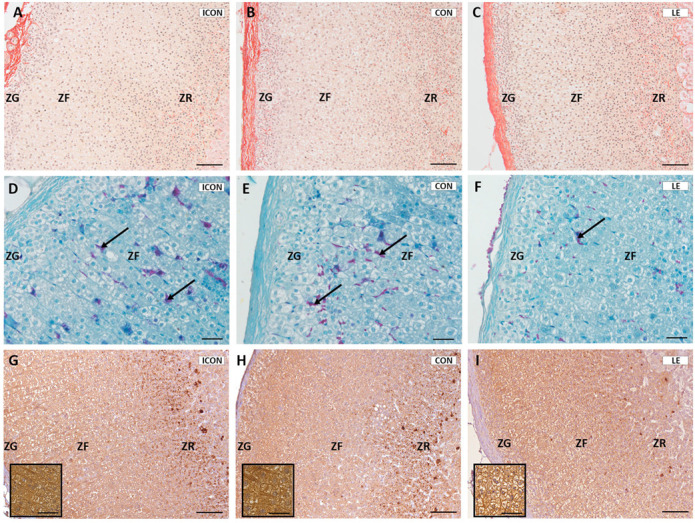
Sirius red- (**A**–**C**), Novelli- (**D**–**F**) and VEGF immune- (**G**–**I**) stained sections of the adrenal gland from aged rats in the intact control group (ICON), the sunflower-oil control group (CON), and the lemon flavonoid extract Eriomin^®^ group (LE); zona glomerulosa (ZG), zona fasciculata (ZF), zona reticularis (ZR); blood vessels (arrows); scale = 50 µm (**A**–**C**,**G**–**I**); scale = 20 µm (**D**–**F** and inserted micrographs).

Gross histological analyses showed clearly recognizable zonation of the adrenal gland in all groups, with no visible pathological changes (Figure 4). The zona glomerulosa (ZG) consisted of spherical cell clusters aligned with radially arranged zona fasciculata (ZF) strands, which connect to the anastomosing network of zona reticularis (ZR) cells in the cortex’s innermost region. Sirius Red staining revealed a dominant presence of collagen fibers in the adrenal capsule, which were also visible in the ZR and medulla. No differences in the quantity or distribution of collagen were observed among the experimental groups (Figure 4A–C).

Examination of the adrenal vasculature using Novelli histological staining revealed purple-stained blood vessels and a capillary network, which were clearly visible against the green-stained background of the adrenal tissue. In the LE-treated group, adrenal sections exhibited fewer prominent stained blood vessels compared to the control groups (Figure 4D–F). Additionally, immunohistochemical analysis of vascular endothelial growth factor (VEGF) showed reduced staining intensity in the adrenal cortex of the LE-treated group compared to the controls (Figure 4G–I).

**Figure 5:**
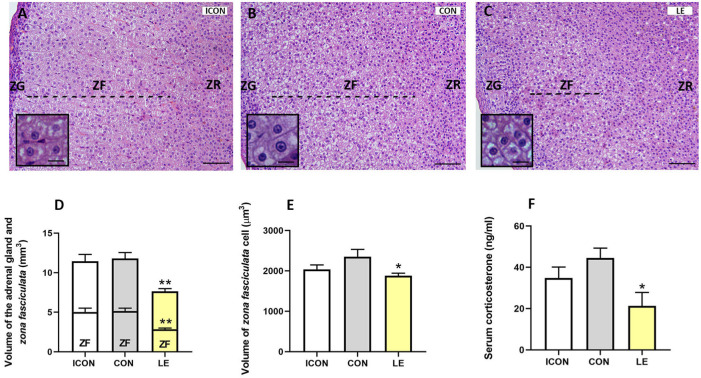
Hematoxylin–eosin-stained sections (**A**–**C**), the absolute volume of the adrenal gland and its zona fasciculata (**D**), the absolute volume of individual cells within the zona fasciculata (**E**), and serum corticosterone concentration (**F**) in the adrenal gland from aged rats in the intact control group (ICON), the sunflower-oil control group (CON) and the lemon flavonoid extract Eriomin^®^ group (LE); scale = 50 µm (**A**–**C**), scale = 20 µm inserted micrographs. Data are presented as means ± SEM (n = six/group); one-way ANOVA test followed by Dunnett’s post hoc test (with CON serving as the control group); * *p* ≤ 0.05, ** *p* ≤ 0.01.

Stereological analysis was performed on hematoxylin–eosin-stained sections (Figure 5A–C). The absolute volume of the adrenal gland was 35% lower in the LE group than in the CON group, and this difference was significant (*p* ≤ 0.01). Similarly, the absolute volume of the ZF was 45% lower (*p* ≤ 0.01) in the LE group (Figure 5D). The volume of individual cells in the ZF was 20% lower in the LE group than in the controls (Figure 5E), and this difference was significant (*p* ≤ 0.05). The serum corticosterone concentration was 52% lower in the LE group than in the CON group (*p* ≤ 0.05) (Figure 5F).

## 3. Discussion

Aging *per se* is associated with increased activity of the PA axis and a decline in negative-feedback inhibition by glucocorticoids, resulting in elevated glucocorticoid levels in blood and tissue in both humans and rodent models [21,22,23]. The present study demonstrated that oral administration of LE significantly reduced PA axis activity in aged rats. The administered oral dose of LE was equivalent to the daily consumption of three to four lemons in humans. Notably, LE treatment led to decreased expression of the *Pomc* gene, which encodes the precursor of ACTH, in the pituitary gland, although no changes were observed in ACTH volume or optical density (i.e., storage within pituitary cells). At the adrenal level, LE treatment resulted in a significant reduction in the mass and absolute volume of the adrenal gland, which was accompanied by a decrease in the absolute volume of the ZF, reduced cell volume, and reduced gland vascularization. Finally, serum corticosterone concentrations significantly decreased following LE treatment.

Eriocitrin, the primary component of LE, along with hesperidin, acts as a partial agonist of peroxisome proliferator-activated receptor gamma (PPARγ), a transcriptional regulator linked to anti-inflammatory, antidiabetic, and antioxidant effects [24,25]. The presence of PPARγ in the hypothalamus, pituitary, and adrenal glands suggests that LE may directly modulate PA axis activity [26,27,28]. Supporting this hypothesis, PPARγ activation inhibits CRH-induced *Pomc* transcription, reduces ACTH and corticosterone levels [29], prevents age-related declines in the expression of glucocorticoid receptors [30], and attenuates axis hyperactivity in diabetic models [31]. Based on these findings, we propose that the observed LE-induced reduction in pituitary *Pomc* gene expression depends on PPARγ activation. The decrease in *Pomc* gene expression despite unchanged intracellular ACTH levels in the pituitary suggests modulation of post-translational processing of POMC in response to current endocrine demands [32]. This implies that even with reduced *Pomc* gene expression, the processing of POMC to ACTH remains stable to maintain hormonal balance, as is reflected by unchanged values of ACTH optical density. Supporting this, ACTH levels also remained unchanged after application of naringenin and hesperetin in the same animal model, which in turn led to increased corticosterone levels [17].

All aspects of corticosteroidogenesis in the adrenal gland, from cholesterol import to conversion to corticosterone, depend on proteins derived from *Pomc* expression [33]. In this study, the observed reduction in *Pomc* gene expression following LE treatment directly corresponded with a decrease in circulating corticosterone levels. The reduction in corticosterone synthesis is further supported by the decrease in both the volume of the ZF and individual cell size. The reduced ZF volume, along with the decreased cell size, correlated directly with diminished corticosterone production, confirming that structural changes in this region reflect impaired steroidogenesis [34].

Additionally, reduced VEGF immunopositivity and adrenal vasculature were observed following LE treatment. These effects may have resulted from decreased steroidogenesis, which reduces the metabolic demand for oxygen and nutrients, thereby diminishing angiogenesis due to the reduction in hormone synthesis [35]. Alternatively, the decrease in vascularization could be attributed to the anti-angiogenic properties of eriocitrin, which has been shown to inhibit phosphorylation of VEGF receptor 2 (VEGFR2) and attenuate downstream signaling cascades mediated by VEGFR2 [36].

The observed reductions in steroidogenesis and vascularization, which were morphologically evidenced by significant decreases in ZF volume and cell volume, resulted in a marked reduction in adrenal volume and mass following LE treatment. These results demonstrate the potential of LE to counteract age-related hyperactivity of the HPA axis, normalize its function, alleviate the negative effects of aging, and reverse age-related dysfunctions in a vital endocrine system. The significance of these findings is further underscored by the fact that LE is a natural product, which is particularly important given that conventional therapeutic drugs often interact with receptors and may cause undesirable side effects.

## 4. Materials and Methods

### 4.1. Animals and Experimental Design

All procedures involving animals complied with Directive 2010/63/EU on the protection of animals used for scientific purposes and were approved by the Ethics Committee for the Use of Laboratory Animals of IBISS, University of Belgrade (approval number 2-12/12).

Twenty-four-month-old male Wistar rats, averaging 450 g in body weight, were obtained from the animal housing facilities of the Institute for Biological Research “Siniša Stanković”, National Institute of the Republic of Serbia (IBISS), Belgrade, Serbia. Animals were housed under standard environmental conditions (12 h light/dark cycle, 22 ± 2 °C) with free access to water and standard rodent food (commercial pellets for rats, Veterinarski zavod, Subotica, Serbia) ad libitum.

For the experimental procedure, the animals were randomly assigned to one of three experimental groups (n = 12): intact control (ICON; received no treatment), sunflower-oil control (CON; orally administered 0.3 mL of sunflower oil) and lemon flavonoid extract Eriomin^®^ (LE; orally administered 0.3 mL of a mixture of lemon extract (40 mg/kg b.w.) and sunflower oil).

The lemon extract Eriomin^®^ (Nature TM, Montclair, CA, USA) is a semi-purified extract composed of citrus flavonoids derived from commercial sources of *Citrus limon* (L.) Burm. f. juice and peel. According to the manufacturer and as verified by high-performance liquid chromatography, the standardized composition includes 70% eriocitrin, 5% hesperidin, 4% naringin, and 1% di-dymin. Additionally, the extract contains 20% fibrous material, including suberin, cutin, lignin, pectin, and cellulose. 

Keeping in mind the percentage composition of individual compounds in Eriomin^®^, it is clear that 40 mg of Eriomin^®^ provides 28 mg of eriocitrin (a flavanone glucoside) and, consequently, 15 mg of its aglycone. This ensures comparability with our previous studies, which used a 15 mg dose of hesperetin or naringenin (also aglycones) in rats [16,18]. The administered dose in rats, calculated allometrically, corresponds to a human equivalent dose of 300 mg/kg per day [37], which falls within the typical supplementary range. Treatments were given daily for four weeks. Based on our earlier research, four weeks of oral administration of citrus flavanones—either as isolated compounds or within an extract—has been shown to be sufficient for the manifestation of effects and the establishment of physiological balance. Furthermore, the chosen dose and regimen are considered safe, as no histopathological changes in the liver were observed in our previous studies [19,20].

### 4.2. Sample Collection and Processing

The animals were humanely euthanized 24 h after the last treatment. Blood was collected, and serum was stored at −80 °C. For histological and stereological analyses, the pituitary and adrenal glands (n = six/group) were excised, weighed, fixed in Bouin’s solution, dehydrated in increasing concentrations of ethanol, cleared in xylene, and embedded in Histowax (Histolab Product Ab, Göteborg, Sweden). The tissue blocks were serially sectioned at 5 μm thickness on a rotary microtome (RM 2125RT Leica Microsystems, Wetzlar, Germany). For PCR analysis, the pituitary tissues (n = six/group) were stored at −80 °C until samples had been collected from all groups.

### 4.3. Histological Staining Procedure

The Novelli (acid fuchsin-light green), Sirius Red, and hematoxylin and eosin (H&E) histological techniques were used for staining sections of the adrenal gland. Novelli staining was applied to assess organ vasculature as previously described [38]. As a result, purple erythrocytes in the blood vessels and capillary network were clearly visible against the green background of the adrenal tissue. Sirius Red staining was used as a method for identification of collagen within adrenal tissue; collagen appeared red or red–orange after staining. Stereological measurements were performed on H&E-stained sections. Within the gland, nucleic acids appeared dark blue, while proteins appeared red-to-orange following the application of routine staining techniques.

### 4.4. Immunohistochemical Staining Procedure

Immunohistochemical (IHC) staining of the pituitary and adrenal sections was performed as previously described [39,40]. In brief, after tissue deparaffinization, endogenous peroxidase activity was blocked by incubation with 0.3% hydrogen peroxide in methanol for 15 min. Reduction of non-specific background staining was achieved by incubation with normal porcine serum (Dakopatts, Glostrup, Denmark; 1:10) for 45 min. ACTH cells were identified by incubation with polyclonal rabbit antisera (donation from Dr. A.F. Parlow, National Institute of Health, Bethesda, MD, USA; 1:1000; AFP-156102789) overnight at 4 °C. Rabbit polyclonal antisera against human vascular endothelial growth factor (VEGF; Abcam, Cambridge, UK; 1:100; abcam^®^ ab46154) were applied (overnight at 4 °C) for the detection of VEGF in the adrenal glands.

### 4.5. Stereological Analysis

All stereological analyses were performed using a newCAST stereological software package (VIS—Visiopharm Integrator System, version 3.2.7.0; Visiopharm, Denmark). Using photomicrographs of ACTH immune-stained sections from three levels of the pituitary for each animal, the volume density of ACTH cells was obtained. Photomicrographs were imported into the Vis program. The volume density of ACTH-ir cells was calculated by dividing the number of points hitting ACTH-ir cells by the number of points hitting the reference space, then multiplying by ×100.

The adrenal gland volume and the volume of the ZF were estimated using Cavalieri’s principle. Sampling of the adrenal gland sections was systematically uniform from a random start. Every 30th section from each tissue block was analyzed. The mean distance between two consecutively studied sections was 150 µm. The volumes of the adrenal gland and ZF were determined according to the following formula:

$$\bar{V}=a\left(p\right)\cdot BA\cdot \sum_{i=1}^{n}Pi$$

where a(p) is the area associated with each sampling point; BA (the block advance) is the mean distance between two consecutively studied sections; n is the number of sections studied for each adrenal gland; and ∑Pi is the sum of points hitting a given target.

The mean individual cell volume in the ZF was estimated using a planar rotator as an unbiased local estimator, and 150–200 cells with nuclei were measured per animal (objective magnification at 100×) [41].

### 4.6. Quantitative Real-Time PCR Analysis

Total RNA from the pituitary gland was isolated using TRIzol (Invitrogen, Carlsbad, CA, USA), following the manufacturer’s instructions. The concentration of RNA was assessed by measuring absorbance on a Nanophotometer^®^ N60 (Implen, Munich, Germany) at 260 nm, while the purity of the samples was determined by A_260_/A_280_ and A_260_/A_230_ ratios. Furthermore, 500 ng of RNA was used for reverse transcription, which was carried out with the High-Capacity cDNA Reverse Transcription Kit (Applied Biosystems by Thermo Fisher Scientific, Waltham, MA, USA). Quantitative real-time PCR (qRT-PCR) analysis was performed using 10-fold-diluted cDNA samples. The QuantStudio^TM^ 3 Real-Time PCR System (Applied Biosystems by Thermo Fisher Scientific) was used for qRT-PCR with Power SYBR^TM^ Green PCR Master Mix (Applied Biosystems by Thermo Fisher Scientific), and primers were designed for specific targets.

The sequences of the primers were as follows: *Pomc*-f: 5′-AGAACGCCATCATCAAGAACG-3, *Pomc*-r: 5′-AGGTCAGGTGCTCTCGCC-3′; *18s rRNA*-f: 5′CGTTGATTAAGTCCCTGCCCTT-3′, *18s rRNA*-r: 5′TCAAGTTCGACCGTCTTCTCAG-3′ (all from Invitrogen, Carlsbad, CA, USA). The expression levels of the target gene was evaluated by comparing its Ct value to the Ct value of *18s rRNA* from the same sample using the 2^–ΔΔCt^ method.

### 4.7. Hormonal Analyses

The blood samples were centrifuged at 3000× *g* for 15 min, and all samples were stored at −80 °C prior to determination of corticosterone concentration using a commercially available corticosterone kit (R&D Systems Inc., Minneapolis, MN, USA). The sensitivity of this kit is 0.047 ng/mL. The plate was read at 450 nm, with wavelength correction set to 570 nm. Corticosterone levels were calculated using a four-parameter logistic curve-fitting program.

### 4.8. Statistical Analysis

Data were analyzed using GraphPad Prism for Windows (v.8, San Diego, CA, USA). No test for outliers was performed. Data distribution was tested using the Kolmogorov–Smirnov normality test. To test the assumption of normality, differences between groups were evaluated using one-way ANOVA followed by Dunnett’s post hoc test. Otherwise, the Kruskal–Wallis test was performed and followed by Dunnett’s post hoc test. Since the comparison between the ICON and CON groups did not show a statistically significant difference, the CON group was used as the control group for all further analyses. Results are expressed as mean values ± SEM, and values of *p* ≤ 0.05, *p* ≤ 0.01, and *p* ≤ 0.001 were considered to be statistically significant.

## 5. Conclusions

In conclusion, the lemon flavonoid extract Eriomin^®^ modulates and restores balance to the pituitary–adrenal axis in aged rats, highlighting its potential biomedical application for addressing age-related dysregulations of the metabolism, cardiovascular system, and other body systems.

## Figures and Tables

**Table 1:** The effects of the lemon flavonoid extract Eriomin^®^ on body mass and pituitary and adrenal weight in aged male rats.

	ICON	CON	LE
Body mass (g)	438.5 ± 47.3	432.8 ± 21.3	440.5 ± 26.1
Absolute pituitary weight (mg)	11.0 ± 1.3	12.9 ± 1.1	12.8 ± 1.5
Relative pituitary weight (%)	2.5 ± 0.3	2.9 ± 0.2	3.0 ± 0.4
Absolute adrenal weight (mg)	26.4 ± 1.4	27.2 ± 1.4	22.2 ± 0.8 ***
Relative adrenal weight (%)	5.5 ± 0.5	6.4 ± 0.2	5.1 ± 0.2 *

Results are presented as means ± SEM (*n* = 12/group). The groups are abbreviated as follows: intact control (ICON), sunflower-oil control (CON) and lemon flavonoid extract Eriomin^®^ (LE); one-way ANOVA test followed by Dunnet’s post hoc test (with CON serving as the main control group). * *p* ≤ 0.05, *** *p* ≤ 0.001.

## Data Availability

The data supporting the conclusions of this article will be made available by the authors upon reasonable request.

## References

[1] Guo J., Huang X., Dou L., Yan M., Shen T., Tang W., Li J. (2022). Aging and Aging-Related Diseases: From Molecular Mechanisms to Interventions and Treatments. Signal Transduct. Target. Ther..

[2] Ageing and Health. https://www.who.int/news-room/fact-sheets/detail/ageing-and-health.

[3] Van Den Beld A.W., Kaufman J.-M., Zillikens M.C., Lamberts S.W.J., Egan J.M., Van Der Lely A.J. (2018). The Physiology of Endocrine Systems with Ageing. Lancet Diabetes Endocrinol..

[4] Stamou M.I., Colling C., Dichtel L.E. (2023). Adrenal Aging and Its Effects on the Stress Response and Immunosenescence. Maturitas.

[5] Gaffey A.E., Bergeman C.S., Clark L.A., Wirth M.M. (2016). Aging and the HPA Axis: Stress and Resilience in Older Adults. Neurosci. Biobehav. Rev..

[6] Goncharova N., Bowden D., Johnson E. (2023). Editorial: The HPA Axis and Aging: Individual Features, Age-Related Pathology. Front. Endocrinol..

[7] Kobayashi N., Machida T., Takahashi T., Takatsu H., Shinkai T., Abe K., Urano S. (2009). Elevation by Oxidative Stress and Aging of Hypothalamic-Pituitary-Adrenal Activity in Rats and Its Prevention by Vitamin E. J. Clin. Biochem. Nutr..

[8] Juszczyk G., Mikulska J., Kasperek K., Pietrzak D., Mrozek W., Herbet M. (2021). Chronic Stress and Oxidative Stress as Common Factors of the Pathogenesis of Depression and Alzheimer’s Disease: The Role of Antioxidants in Prevention and Treatment. Antioxidants.

[9] Signorello M.G., Ravera S., Leoncini G. (2024). Oxidative Stress Induced by Cortisol in Human Platelets. Int. J. Mol. Sci..

[10] Godonu S.S., Francis-Lyons N. (2025). Role Of Cortisol in The Synthesis of Glutamate During Oxidative Stress. Int. J. Sci. Res. Technol..

[11] Ribeiro C.B., Ramos F.M., Manthey J.A., Cesar T.B. (2019). Effectiveness of Eriomin^®^ in Managing Hyperglycemia and Reversal of Prediabetes Condition: A Double-blind, Randomized, Controlled Study. Phytother. Res..

[12] Mohammadi N., Dos Santos Lima A., Azevedo L., Granato D. (2024). Bridging the Gap in Antioxidant Activity of Flavonoids: Correlating the Oxidation of Human Plasma with Chemical and Cellular Assays. Curr. Res. Food Sci..

[13] Yao L., Liu W., Bashir M., Nisar M.F., Wan C. (2022). (Craig) Eriocitrin: A Review of Pharmacological Effects. Biomed. Pharmacother..

[14] Ji M., Deng Z., Rong X., Li R., You Z., Guo X., Cai C., Zhao Y., Gao P., Cao G. (2022). Naringenin Prevents Oxidative Stress and Inflammation in LPS-Induced Liver Injury through the Regulation of LncRNA-mRNA in Male Mice. Molecules.

[15] Madureira M.B., Concato V.M., Cruz E.M.S., Bitencourt De Morais J.M., Inoue F.S.R., Concimo Santos N., Gonçalves M.D., Cremer De Souza M., Basso Scandolara T., Fontana Mezoni M. (2023). Naringenin and Hesperidin as Promising Alternatives for Prevention and Co-Adjuvant Therapy for Breast Cancer. Antioxidants.

[16] Miler M., Živanović J., Ajdžanović V., Oreščanin-Dušić Z., Milenković D., Konić-Ristić A., Blagojević D., Milošević V., Šošić-Jurjević B. (2016). Citrus Flavanones Naringenin and Hesperetin Improve Antioxidant Status and Membrane Lipid Compositions in the Liver of Old-Aged Wistar Rats. Exp. Gerontol..

[17] Miler M., Živanović J., Kovačević S., Vidović N., Djordjevic A., Filipović B., Ajdžanović V. (2024). Citrus Flavanone Effects on the Nrf2-Keap1/GSK3/NF-κB/NLRP3 Regulation and Corticotroph-Stress Hormone Loop in the Old Pituitary. Int. J. Mol. Sci..

[18] Miler M., Živanović J., Ajdžanović V., Milenkovic D., Jarić I., Šošić-Jurjević B., Milošević V. (2020). Citrus Flavanones Upregulate Thyrotroph Sirt1 and Differently Affect Thyroid Nrf2 Expressions in Old-Aged Wistar Rats. J. Agric. Food Chem..

[19] Šošić-Jurjević B., Borković-Mitić S., Pavlović S., Vlahović D., Miler M., Cesar T., Ajdžanović V., Milenkovic D., Stellaard F., Trifunović S. (2024). Lemon Flavonoid Extract Eriomin Improves Pro/Antioxidant Status and Interferes with Cholesterol Metabolism without Affecting Serum Cholesterol Levels in Aged Rats. Int. J. Mol. Sci..

[20] Miler M., Živanović J., Ajdžanović V., Milenković D., Cesar T., Filipović M.R., Milošević V. (2024). Lemon Extract Reduces the Hepatic Oxidative Stress and Persulfidation Levels by Upregulating the Nrf2 and Trx1 Expression in Old Rats. BioFactors.

[21] Garrido P., De Blas M., Del Arco A., Segovia G., Mora F. (2012). Aging Increases Basal but Not Stress-Induced Levels of Corticosterone in the Brain of the Awake Rat. Neurobiol. Aging.

[22] Tesic V., Ciric J., Jovanovic Macura I., Zogovic N., Milanovic D., Kanazir S., Perovic M. (2021). Corticosterone and Glucocorticoid Receptor in the Cortex of Rats during Aging—The Effects of Long-Term Food Restriction. Nutrients.

[23] Trifunović S., Manojlović-Stojanoski M., Ajdžanović V., Nestorović N., Ristić N., Medigović I., Milošević V. (2012). Genistein Stimulates the Hypothalamo-Pituitary-Adrenal Axis in Adult Rats: Morphological and Hormonal Study. Histol. Histopathol..

[24] Gamo K., Shiraki T., Matsuura N., Miyachi H. (2014). Mechanism of Peroxisome Proliferator-Activated Receptor Gamma (PPARγ) Transactivation by Hesperetin Glucuronides Is Distinct from That by a Thiazolidine-2,4-Dione Agent. Chem. Pharm. Bull..

[25] Gangadhariah M., Pardhi T., Ravilla J., Chandra S., Singh S.A. (2023). Citrus Nutraceutical Eriocitrin and Its Metabolites Are Partial Agonists of Peroxisome Proliferator-Activated Receptor Gamma (PPARγ): A Molecular Docking and Molecular Dynamics Study. J. Biomol. Struct. Dyn..

[26] Qi W., Zhong L., Li X., Li G., Liu Z., Hu J., Chen N. (2012). Hyperglycemia Induces the Variations of 11 β -Hydroxysteroid Dehydrogenase Type 1 and Peroxisome Proliferator-Activated Receptor- γ Expression in Hippocampus and Hypothalamus of Diabetic Rats. J. Diabetes Res..

[27] Wiesner G., Morash B., Ur E., Wilkinson M. (2004). Food Restriction Regulates Adipose-Specific Cytokines in Pituitary Gland but Not in Hypothalamus. J. Endocrinol..

[28] Zaripheh S., Nara T.Y., Nakamura M.T., Erdman J.W. (2006). Dietary Lycopene Downregulates Carotenoid 15,15′-Monooxygenase and PPAR-γ in Selected Rat Tissues. J. Nutr..

[29] Mannelli M., Cantini G., Poli G., Mangoni M., Nesi G., Canu L., Rapizzi E., Borgogni E., Ercolino T., Piccini V. (2010). Role of the PPAR-γ System in Normal and Tumoral Pituitary Corticotropic Cells and Adrenal Cells. Neuroendocrinology.

[30] Escribano L., Simón A.-M., Pérez-Mediavilla A., Salazar-Colocho P., Río J.D., Frechilla D. (2009). Rosiglitazone Reverses Memory Decline and Hippocampal Glucocorticoid Receptor Down-Regulation in an Alzheimer’s Disease Mouse Model. Biochem. Biophys. Res. Commun..

[31] Torres R.C., Magalhaes N.S., Silva P., Martins M.A., Carvalho V.F. (2016). Activation of PPAR-γ Reduces HPA Axis Activity in Diabetic Rats by up-Regulating PI3K Expression. Exp. Mol. Pathol..

[32] Harno E., Gali Ramamoorthy T., Coll A.P., White A. (2018). POMC: The Physiological Power of Hormone Processing. Physiol. Rev..

[33] Karpac J., Czyzewska K., Kern A., Brush R.S., Anderson R.E., Hochgeschwender U. (2008). Failure of Adrenal Corticosterone Production in *Pomc*-Deficient Mice Results from Lack of Integrated Effects of *Pomc* Peptides on Multiple Factors. Am. J. Physiol. Endocrinol. Metab..

[34] Conran R.M., Nickerson P.A. (1982). Atrophy of the zona fasciculata in the adrenal cortex of thyroparathyroidectomized rats: A quantitative study. Am. J. Anat..

[35] Hinson J.P., Vinson G.P., Kapas S., Teja R. (1991). The Relationship Between Adrenal Vascular Events and Steroid Secretion: The Role of Mast Cells and Endothelin. J. Steroid Biochem. Mol. Biol..

[36] Baek J.-Y., Kwak J.-E., Ahn M.-R. (2024). Eriocitrin Inhibits Angiogenesis by Targeting VEGFR2-Mediated PI3K/AKT/mTOR Signaling Pathways. Nutrients.

[37] Nair A., Jacob S. (2016). A Simple Practice Guide for Dose Conversion between Animals and Human. J. Basic Clin. Pharm..

[38] Šošić-Jurjević B., Filipović B., Renko K., Miler M., Trifunović S., Ajdžanovič V., Kӧhrle J., Milošević V. (2015). Testosterone and Estradiol Treatments Differently Affect Pituitary-Thyroid Axis and Liver Deiodinase 1 Activity in Orchidectomized Middle-Aged Rats. Exp. Gerontol..

[39] Zivkovic A., Trifunovic S., Savic D., Milosevic K., Lavrnja I. (2024). Experimental Autoimmune Encephalomyelitis Influences GH-Axis in Female Rats. Int. J. Mol. Sci..

[40] Šošić-Jurjević B., Ajdžanović V., Filipović B., Trifunović S., Jarić I., Ristić N., Milošević V. (2016). Functional Morphology of Pituitary -Thyroid and -Adrenocortical Axes in Middle-Aged Male Rats Treated with Vitex Agnus Castus Essential Oil. Acta Histochem..

[41] Jensen E.B.V., Gundersen H.J.G. (1993). The Rotator. J. Microsc..

